# Hsa_circ_0001869 promotes NSCLC progression via sponging miR-638 and enhancing FOSL2 expression

**DOI:** 10.18632/aging.104037

**Published:** 2020-11-20

**Authors:** Pei Xu, Lei Wang, Xiao Xie, Fengqing Hu, Qi Yang, Rui Hu, Lianyong Jiang, Fangbao Ding, Ju Mei, Jianhui Liu, Haibo Xiao

**Affiliations:** 1 Department of Cardiothoracic Surgery, Xinhua Hospital, Shanghai Jiaotong University School of Medicine, Shanghai 200092, China; 2 Department of Anesthesiology, Shanghai Tongji Hospital, Tongji Medical School, Tongji University, Shanghai 200065, China

**Keywords:** hsa_circ_0001869, NSCLC, miR-638, FOSL2, luciferin report analysis

## Abstract

Accumulating studies suggest that circular RNAs (circRNAs) function as key regulators in human cancers. We found that hsa_circ_0001869 participated in non-small cell lung cancer (NSCLC) progression. However, its expression and function during NSCLC remain unknown. The data advised that hsa_circ_0001869 expression was increased in NSCLC cell lines and tissues. High hsa_circ_0001869 expression had negatively correlation with the NSCLC patients prognosis. Bioinformatics and luciferase report analyses confirmed that miR-638 and FOSL2 were hsa_circ_0001869 downstream target. hsa_circ_0001869 downregulation decreased tumor proliferation, invasion and migration by promoting miR-638 expression and decreasing FOSL2 expression. As a result of overexpression of FOSL2 or silencing of miR-638, the recovery of proliferation, migration, and invasion after hsa_circ_0001869 silencing. Overexpression of FOSL2 also led to recovery of migration, invasion and proliferation after upregulation of miR-638. *In vivo* studies confirmed that overexpression of FOSL2 or silencing of miR-638 led to the recovery of tumor growth ability regarding A549 cells after hsa_circ_0001869 knockdown. Present investigation discovered that hsa_circ_0001869 enhanced NSCLC progression via sponging miR-638 and promoting FOSL2 expression. hsa_circ_0001869 downregulation suppressed tumor growth and invasion ability.

## INTRODUCTION

NSCLC is a main reason concerning lung cancer-related mortality [1, 2], which contributes to almost 85% of the lung cancer cases. It is essential to improve patient survival through early diagnosis as well as treatment. Nevertheless, the mechanisms regarding lung cancer progression has not yet been completely elucidated [3, 4]. Non-coding RNAs (ncRNAs) participated in various biological processes can be identified based on the recent advances in RNA studies. Circular RNAs (circRNAs) belong to RNAs, which are important for human malignant tumors [5, 6]. But, it is still uncertain how the biological of circRNAs in human diseases function, especially in cancer pathogenesis. Researches indicate that some circRNAs possess microRNA (miRNA) binding sites can be processed by some circRNAs and miRNA downstream targets can also be regulated by circRNAs through sponging and arresting the activity of miRNAs [7–9].

CircRNAs belong to the class of RNAs that are recently founded and widely expressed in eukaryotic cells. The understanding of circRNAs has remarkably improved result in the rapid development of high-throughput sequencing and bioinformatics [10–14]. Many circRNAs are related to the cancer initiation and progression. For example, the circRNA circ-PRMT5 facilitates NSCLC proliferation by upregulating EZH2 through sponging miR-377/382/498 [15]. The circRNA ZFR accelerates NSCLC progression by playing a role as miR-101-3p sponge to promote CUL4B expression [16]. The circRNA circPIP5K1A advanced NSCLC metastasis and proliferation via miR-600/HIF-1α regulation [17]. Recently, there is a hypothesis that competing endogenous RNAs (ceRNAs), including lncRNAs, mRNAs, and pseudogenes, could have the ability of combining with and modulating each other through binding to response elements (MREs) of microRNA [18]. Several researches have indicated that circRNAs can act as ceRNAs to hender miRNAs from approaching their target genes. In this research, we have some findings about hsa_circ_0001869 that had abnormal expression in patients with NSCLC. Nevertheless, it is still uncertain regarding to hsa_circ_0001869 function and regulatory mechanism as ceRNA in NSCLC progression. Therefore, our team inspected the biological function of hsa_circ_0001869 and the underlying molecular mechanisms in NSCLC development from multiple perspectives. Results provide unusual clues about the NSCLC biomarkers identification.

## RESULTS

### High hsa_circ_0001869 expression predicted unfavorable NSCLC prognosis

**Figure 1:**
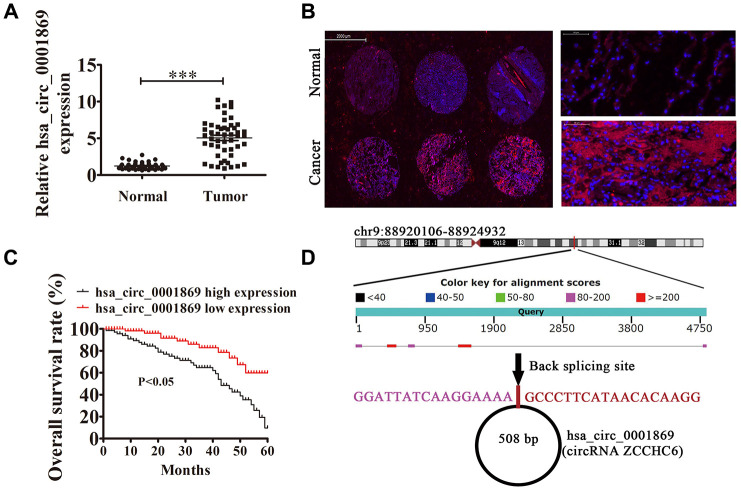
**High expression of hsa_circ_0001869 predicted unfavorable prognosis of non-small cell lung cancer (NSCLC).** (**A**) qRT-PCR assay of hsa_circ_0001869 in 50 paired NSCLC tumor tissues and adjacent nontumor tissues. Data are expressed as mean ± SD. ^***^P < 0.001 vs. normal controls. (**B**) Fluorescent *in situ* hybridization indicates subcellular localization of hsa_circ_0001869. (**C**) Prognostic significance of hsa_circ_0001869 expression for hsa_circ_0001869 patients was performed with FISH using the median value as the cutoff. Observation time was 60 months. (**D**) Genomic loci of *ZCCHC6* and hsa_circ_0001869. Red, back splicing.

**Figure 2:**
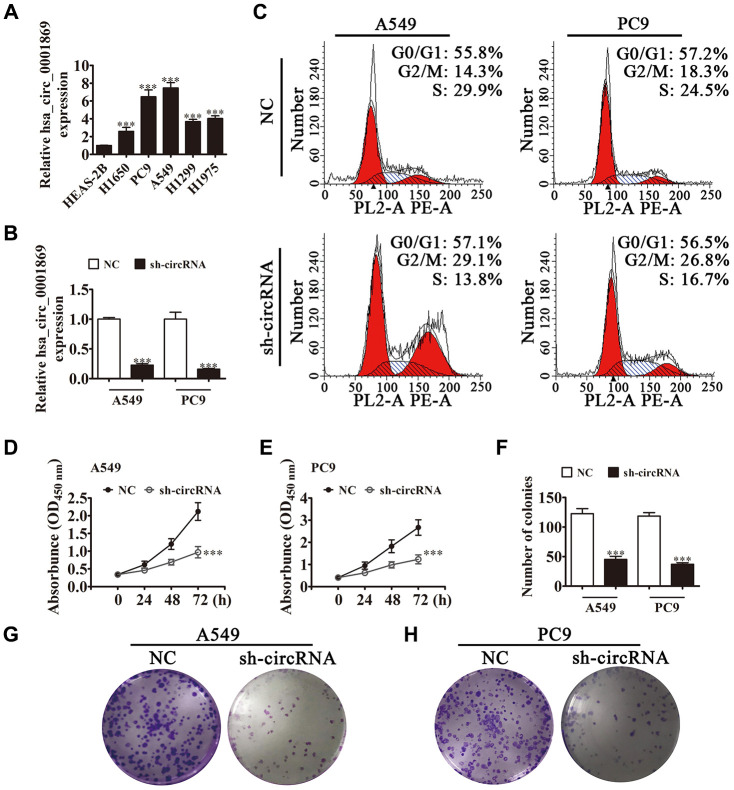
**Hsa_circ_0001869 expression increased in NSCLC cell lines and knockdown of hsa_circ_0001869 expression suppressed cell proliferation.** (**A**) qRT-PCR for hsa_circ_0001869 expression in A549, PC9, H1299, H1975, and H1650 tumor cells and the normal lung epithelial cell line HEAS-2B. n = 3. Data are expressed as mean ± SD. ^***^P < 0.001 vs. normal group. (**B**) qRT-PCR analysis of expression of hsa_circ_0001869 in A549 and PC9 cells after knockdown of hsa_circ_0001869 (sh-circRNA). n = 3. Data are expressed as mean ± SD. ^***^P < 0.001 vs. NC. (**C**) Cell cycle distribution by flow cytometry after PI staining. (**D** and **E**) CCK8 detection for the proliferation of A549 (**D**) and PC9 (**E**) cells with or without hsa_circ_0001869 silencing. n = 3. Data are expressed as mean ± SD. ^***^P < 0.001 vs. NC. (**F**–**H**) Colony formation assay revealed colony-forming ability of A549 and PC9 cells. n = 3. Data are expressed as mean ± SD. ^***^P < 0.001 vs. NC.

qRT-PCR verified that hsa_circ_0001869 expression increased in human NSCLC tissues comparing with adjacent normal tissues (Figure 1A). We chose 150 human NSCLC and adjacent normal tissue pairs for hsa_circ_0001869 FISH assay, which illustrated that hsa_circ_0001869 was predominately localized to cytoplasm (Figure 2B). We divided samples into two groups following expression levels: relatively high expression (higher than adjacent normal tissues; n = 68) and relatively low expression (lower than adjacent normal tissues; n = 82). No relationship was observed between hsa_circ_0001869 expression and clinical factors of gender (male or female) or age (> 60 and ≤60 years). Significant differences were found for lymph node metastasis (positive and negative), TNM stage (I/II or III/IV, high), and tumor size (≤3 and >3 cm) (Table 1). Gehan-Breslow-Wilcoxon test survival curves validated that NSCLC with high hsa_circ_0001869 expression predicted poor overall survival (Figure 1C), suggesting that hsa_circ_0001869 affected NSCLC progression. Hsa_circ_0001869 originated from circularizing five exons from *ZCCHC6* gene, which is located at chr9:88920106-88924932. *ZCCHC6* has 4826 bp, and the spliced mature circRNA is 508 bp (Figure 1D).

**Table 1:** The clinic-pathological factors of 150 NSCLC patients.

**Characteristics**	**Numbers**	**Expression of hsa_circ_0001869**		***P* value**
		**Low (N = 82)**	**High (N = 68)**	
**Sex**				0.103
male	79	48	31	
female	71	34	37	
**Age**				0.082
≤60	79	43	36	
>60	71	39	32	
**TNM stage**				0.026
I and II	91	71	20	
III and IV	59	11	48	
**Lymph node metastasis**				0.018
negative	108	74	34	
positive	42	8	34	
**Tumor size**				0.023
≤ 3 cm	88	63	15	
> 3 cm	62	9	53	

### hsa_circ_0001869 knockdown suppressed NSCLC proliferation

qRT-PCR verified that hsa_circ_0001869 expression increased in NSCLC cell lines PC9, A549, H1299, H1650 and H1975 comparing with that in normal lung epithelial cells HEAS-2B (Figure 2A). A549 and PC9 cells had the highest hsa_circ_0001869 expression, which were picked up for next-step studies. qRT-PCR results also revealed that hsa_circ_0001869 expression decreased significantly after hsa_circ_0001869 depletion in PC9 and A549 cells (Figure 2B). Cell cycle distribution analyses demonstrated that S-phase cell proportion decreased significantly and that of G2/M-phase cells increased after hsa_circ_0001869 depletion, thus inferring cell cycle arrest at G2/M phase (Figure 2C). CCK8 (Figure 2D and 2E) and colony formation assays (Figure 2F–2H) demonstrated that hsa_circ_0001869 silencing suppressed A549 and PC9 cell proliferation.

**Figure 3:**
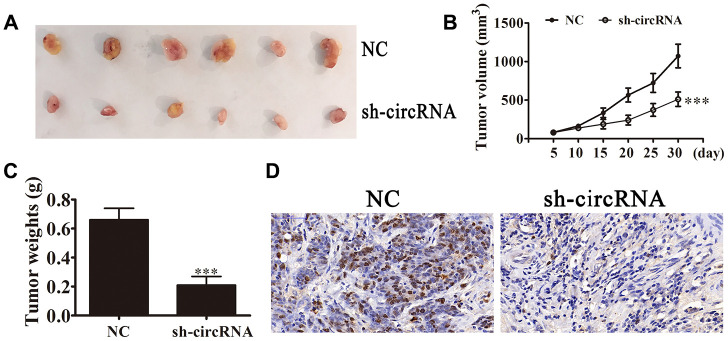
**hsa_circ_0001869 silencing suppressed tumor growth of xenografts in nude mice.** (**A** and **B**) Photographs of tumors and curves for tumor volume growth (**B**) for nude mice. n = 6. Data are expressed as mean ± SD. ^***^P < 0.001 vs. NC. (**C**) Tumor weight. n = 6. Data are expressed as mean ± SD. ^***^P < 0.001 vs. NC. (**D**) Ki-67 staining of tumor tissue sections.

We used *in vivo* xenograft mouse models to investigate the hsa_circ_0001869 effects towards tumor development. We inoculated nude mice using hsa_circ_0001869-silenced (sh-circRNA) or negative control (NC) A549 cells. After 30 d, we harvested xenografts (Figure 3A). We observed smaller tumor sizes in mice injected with sh-circRNA A549 cells, and discovered larger xenograft sizes in mice inoculated with WT cells. Xenografts developed more slowly in sh-circRNA group in terms of both volume (Figure 3B) and weight (Figure 3C). IHC suggested that Ki-67 expression levels reduced in A549 sh-circRNA group (Figure 3D). These results illustrated that hsa_circ_0001869 knockdown suppressed the NSCLC proliferation *in vitro* and *in vivo*.

### hsa_circ_0001869 knockdown decreased NSCLC invasion ability *in vivo* and *in vitro*

**Figure 4:**
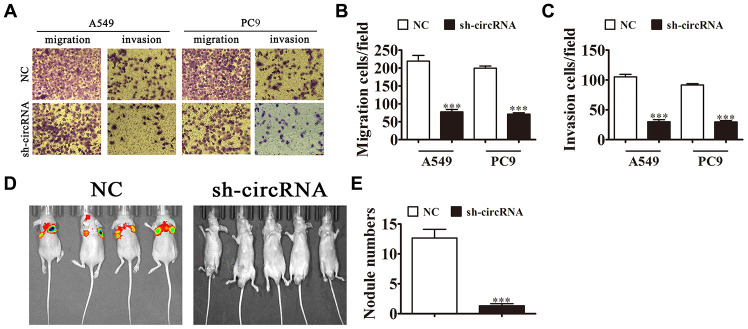
**Knockdown of hsa_circ_0001869 decreased the invasion ability of NSCLC *in vitro* and *in vivo*.** (**A**–**C**) Cell migration and invasion were determined in A549 and PC9 cells using the Transwell^®^ assay. n = 3. Data are expressed as mean ± SD. ^***^P < 0.001 vs. NC. (**D**) Live imaging of the effects of hsa_circ_0001869 on metastasis of A549 cells 30 days after intravenous tail injection. (**E**) Number of lung metastatic nodules by group. n = 6. Data are expressed as mean ± SD. ^***^P < 0.001 vs. NC.

We investigated hsa_circ_0001869 effect on NSCLC invasiveness. *In vitro* experiments showed that hsa_circ_0001869 knockdown suppressed cell invasion and migration in A549 and PC9 cells in Transwell assay (Figure 4A–4C). We injected intravenously A549 cells with or not hsa_circ_0001869 knockdown into mice tails and studied them at 30 days after injection. The live imaging analysis advised that A549 cell metastasis ability decreased after hsa_circ_0001869 silencing (Figure 4D). The number of lung metastatic nodules decreased in hsa_circ_0001869 silenced group (Figure 4E). This result suggested that knockdown of hsa_circ_0001869 suppressed NSCLC invasion ability *in vitro* and *in vivo*.

### Hsa_circ_0001869 binds directly to miR-638 to facilitate NSCLC progression

**Figure 5:**
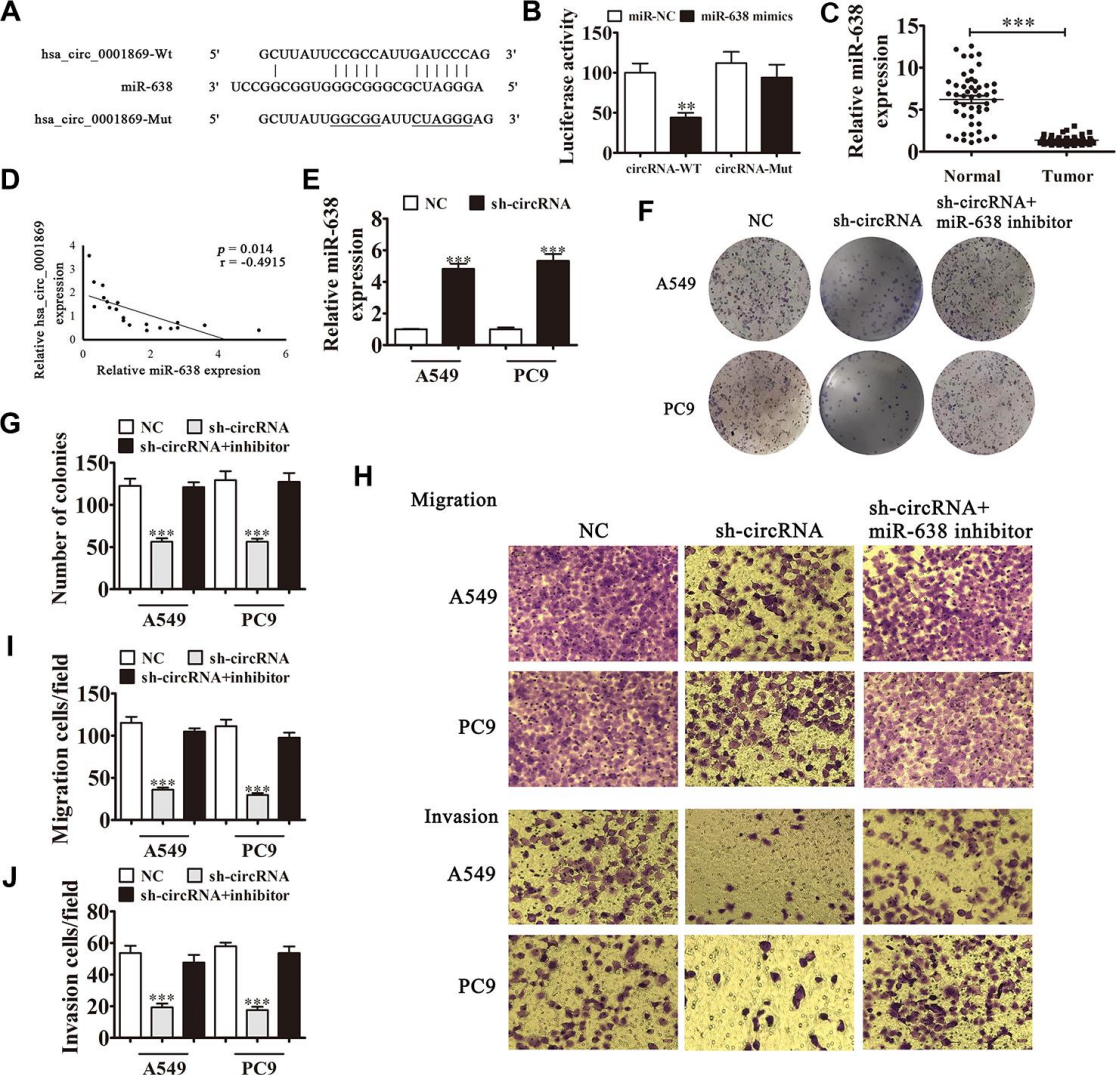
**hsa_circ_0001869 binds directly to miR-638 to facilitate NSCLC progression.** (**A**) Predicted binding sites of miR-638 in hsa_circ_0001869. Mutated (Mut) version of hsa_circ_0001869 is shown. (**B**) Relative luciferase activity was determined 48 h after transfection with miR-638 mimic/normal control (NC) or with hsa_circ_0001869 wild-type/Mut in HEK293T cells. n = 3. Data are expressed as mean ± SD. ^***^P < 0.001. (**C**) miR-638 expression was examined in NSCLC tissues and matched peritumor samples using qRT-PCR. Expression level of miR-638 was significantly lower in NSCLC tissues than in peritumor samples. Data are expressed as mean ± SD, n = 20. ^***^P < 0.001 vs. normal tissues. (**D**) Significant negative correlation was observed between hsa_circ_0001869 and miR-638 in NSCLC tissues: n = 20, P = 0.014. (**E**) qRT-PCR was used to investigate miR-638 levels in hsa_circ_0001869-silenced A549 and PC9 cells. Data are expressed as mean ± SD. ^***^P < 0.001 vs. NC. (**F** and **G**) Colony formation assay was used to determine the colony-forming ability of A549 and PC9 cells after knockdown of hsa_circ_0001869 with or without silencing miR-638. Data are expressed as mean ± SD. ^***^P < 0.001 vs. NC. (**H**–**J**) Cell migration and invasion were determined in A549 and PC9 cells using the Transwell^®^ assay. n = 3. Data are expressed as mean ± SD. ^***^P < 0.001 vs. NC.

Bioinformatics analysis showed that miR-638 was hsa_circ_0001869 downstream target. To confirm the correlation between miR-638 and hsa_circ_0001869, we built WT or MUT hsa_circ_0001869 sequences containing miR-638 binding sequence in luciferase reporter vector (Figure 5A). We transfected vector into 293T cells with or not an miR-638 mimic. The luciferase reporter analysis revealed that miR-638 inhibited luciferase activity in WT but not in MUT cell lines (Figure 5B), advising that miR-638 was a hsa_circ_0001869 target. qRT-PCR results also illustrated that miR-638 expression reduced in NSCLC tissues comparing to adjacent normal tissues (Figure 5C). We observed negative correlation between hsa_circ_0001869 expression and miR-638 levels in NSCLC tissues (Figure 5D). *In vitro* experiments also verified that hsa_circ_0001869 knockdown significantly increased miR-638 levels in A549 and PC9 cells (Figure 5E). A colony formation assay (Figure 5F and 5G) and Transwell assay (Figure 5H–5J) illustrated that the miR-638 downregulation resulted in recovery concerning invasion, migration and proliferation ability of PC9 and A549 cells after hsa_circ_0001869 knockdown. This result suggested that hsa_circ_0001869 enhanced NSCLC proliferation and invasion via sponging miR-638.

### FOSL2 is FOSL2/miR-638 axis downstream target

**Figure 6:**
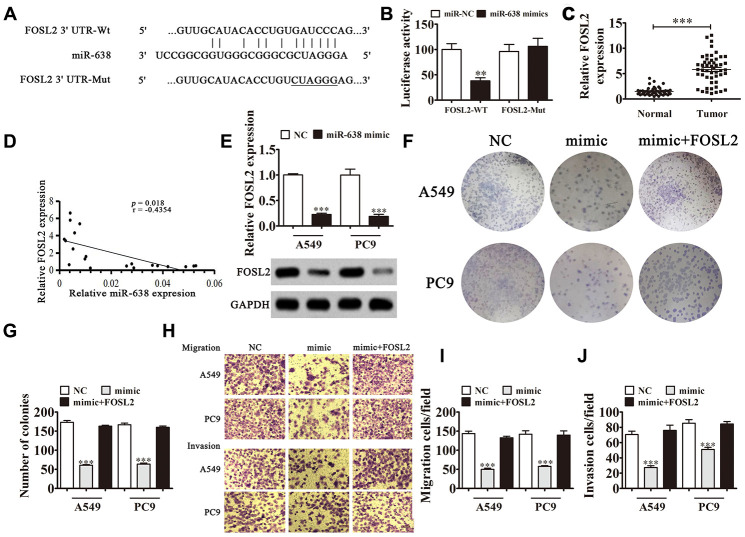
**FOSL2 is a downstream target of the FOSL2/miR-638 axis.** (**A**) Predicted binding sites in 3′-UTR-FOSL2 for miR-638. Mutated (Mut) version of 3′-UTR-FOSL2 is shown. (**B**) Relative luciferase activity was determined 48 h after transfection with miR-638 mimic/normal control (NC) or 3′-UTR-FOSL2 wild-type/Mut in HEK293T cells. Data are expressed as mean ± SD. ^***^P < 0.001. (**C**) FOSL2 expression was examined in NSCLC tissues and matched peritumor samples using qRT-PCR. FOSL2 expression was significantly increased in NSCLC tissues compared to that in peritumor samples. Data are expressed as mean ± SD, n = 50. ^***^P < 0.001 vs. normal tissues. (**D**) Significant negative correlation was observed between FOSL2 and miR-638 in NSCLC tissues: n = 50, P = 0.018. (**E**) qRT-PCR was used to investigate FOSL2 levels in miR-638-overexpressing A549 and PC9 cells. n = 3. Data are expressed as mean ± SD. ^***^P < 0.001 vs. NC. (**F** and **G**) Colony formation assay revealed the colony-forming ability of A549 and PC9 cells after overexpression of miR-638 with or without FOSL2 overexpression (LV-FOSL2 or FOSL2). n = 3. Data are expressed as mean ± SD. ^***^P < 0.001 vs. NC. (**H**–**J**) Cell migration and invasion were determined for A549 and PC9 cells using the Transwell^®^ assay. Data are expressed as mean ± SD. ^***^P < 0.001 vs. NC.

Bioinformatics analysis showed that FOSL2 was miR-638 downstream target. To confirm the correlation between miR-638 and FOSL2, we constructed MUT or WT 3'UTR-FOSL2 sequences including miR-638 binding sequence in luciferase reporter vector (Figure 6A). We transfected vector into 293T cells with or without an miR-638 mimic. The luciferase reporter analysis showed that miR-638 inhibited luciferase activity in WT but not in MUT cell lines (Figure 6B), advising that FOSL2 was a miR-638 target. qRT-PCR results verified that FOSL2 expression enhanced in NSCLC tissues comparing with adjacent normal tissues (Figure 6C). We observed negative correlation between miR-638 expression and FOSL2 level in NSCLC tissues (Figure 6D). *In vitro* experiments validated that the miR-638 overexpression decreased FOSL2 levels significantly in A549 and PC9 cells (Figure 6E). A colony formation assay (Figure 6F and 6G) and Transwell assay (Figure 6H–6J) revealed that miR-638 overexpression decreased migration, proliferation, and invasion in PC9 and A549 cells. FOSL2 overexpression resulted in proliferation, invasion and migration recovery in PC9 and A549 cells after miR-638 upregulation. This result suggested that miR-638 expression suppressed NSCLC cell invasion and proliferation through targeting FOSL2.

**Figure 7:**
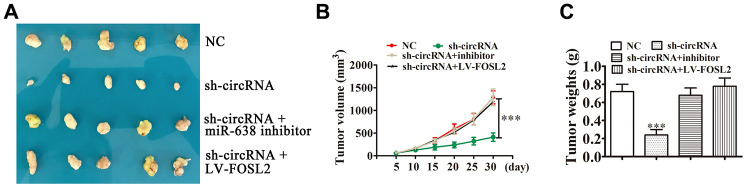
**hsa_circ_0001869 facilitated NSCLC progression *in vivo* by targeting miR-638 and upregulating FOSL2 expression.** (**A**) Xenograft tumors in nude mice from four treatment groups (sh-NC, sh-circRNA, sh-circRNA + miR-638 inhibitor, and sh-circRNA + LV-FOSL2) after subcutaneous injection of A549 cells. (**B**) Xenograft volumes from four treatment groups measured at the indicated time points. n = 5. Data are expressed as mean ± SD. ^***^P < 0.001 vs. (**C**) Xenograft weights were compared among the groups. Data are expressed as mean ± SD. ^***^P < 0.001 vs. sh-NC.

*In vivo* xenograft mouse models developed through A549 cells illustrated that miR-638 downregulation or FOSL2 upregulation resulted in tumor growth ability recovery regarding A549 cells after hsa_circ_0001869 knockdown (Figure 7A–7C). This result suggested that the FOSL2/miR-638 axis was hsa_circ_0001869 downstream target. Hsa_circ_0001869 enhanced NSCLC progression via sponging miR-638 and promoting FOSL2 expression.

## DISCUSSION

CircRNA expression profiling is indispensable to identify novel tumor suppressors and oncogenic circRNAs and to elucidate the functions and mechanisms [19]. In current study, we discovered that hsa_circ_0001869 expression promoted in NSCLC tissues and cell lines. High hsa_circ_0001869 expression predicted poor prognosis. Cell cycle changes are usual in nearly all cancers. Current investigation found that hsa_circ_0001869 downregulation arrested the cell cycle at G2/M phase. hsa_circ_0001869 knockdown suppressed NSCLC cell migration, proliferation, and invasion. *In vivo* experiments also showed that hsa_circ_0001869 silencing inhibits A549 cell tumor growth. This result suggested that hsa_circ_0001869 was important for NSCLC progression.

Circular RNA profiling of different cancers has been extensively studied. CircTP63 competitively binds to miR-873-3p and prevents it from decreasing the FOXM1 level, which upregulates CENPB and CENPA so as to facilitate cell cycle progression [20]. hsa_circ_0001946 inhibits lung cancer progression and mediates cisplatin sensitivity in NSCLC through nucleotide excision repair signaling pathway [11]. CircAGFG1 sponges miR-203 to enhance epithelial-mesenchymal transition and metastasis regarding NSCLC through ZNF281 expression upregulation [12]. The regulatory hsa_circ_0001869 mechanism in NSCLC is still unclear.

CircRNAs are noncoding RNAs that participate in tumor development, mainly by sponging miRNA [13]. By using bioinformatics analysis, we discovered that miR-638 was hsa_circ_0001869 downstream target. A luciferase assay validated that miR-638 interacted with hsa_circ_0001869. hsa_circ_0001869 downregulation significantly increased miR-638 expression. miR-638 expression decreased in NSCLC tissues. We observed negative correlation between miR-638 and hsa_circ_0001869. Former investigations showed that miR-638 has anticancer effect [21–23]. miR-638 downregulation enhances breast cancer progression, which is associated with patients breast cancer prognosis [24]. miR-638 inhibits cervical cancer metastasis via the Wnt/β-catenin signaling pathway, which associates with the patient cervical cancer prognosis [25]. We found that miR-638 was important for regulating the progression of NSCLC. miR-638 downregulation caused the recovery of NSCLC proliferation, migration and invasion ability after hsa_circ_0001869 knockdown. This result suggested that hsa_circ_0001869 enhanced NSCLC cell invasion and proliferation via sponging miR-638.

To further determine hsa_circ_0001869/miR-638 axis regulatory mechanism for NSCLC progression, we conducted bioinformatics analysis and found that FOS-like antigen 2 (FOSL2) was the miR-638 downstream target. A luciferase assay validated that miR-638 interacted with FOSL2 3′-UTR. miR-638 overexpression significantly decreased FOSL2 expression. FOSL2 expression increased in NSCLC tissues. We observed negative correlation between FOSL2 and miR-638. Previous studies showed that FOSL2 expression promotes proliferation, migration, and invasion of cancers, including osteosarcoma [26], hepatocellular carcinoma [27], and colon cancer [28, 29]. We discovered that FOSL2 was important for regulating NSCLC progression. Downregulation of FOSL2 led to the recovery of NSCLC proliferation, migration, and invasion ability after hsa_circ_0001869 knockdown. Overexpression of FOSL2 also caused recovery of NSCLC migration, proliferation, and invasion ability after upregulation of miR-638. This result advised that FOSL2 was the FOSL2/miR-638 axis downstream target.

In conclusion, an increase in hsa_circ_0001869 expression in NSCLC closely correlated to NSCLC occurrence and development. We showed that hsa_circ_0001869 directly targeted miR-638 through the FOSL2 expression upregulation. hsa_circ_0001869 downregulation suppressed NSCLC progression via promoting miR-638 and decreasing FOSL2 expression. This result provided a new NSCLS treatment target, which thus deserves more investigation.

## MATERIALS AND METHODS

### Tissue samples

We obtained 150 fresh NSCLC tissues and paired adjacent noncancerous lung tissues with patient informed consent in the Xinhua Hospital affiliated to Shanghai Jiaotong University, China. We evaluated pathological and patient histological features with NSCLC based upon Revised International System for Staging Lung Cancer. Patients did not receive any chemotherapy or radiotherapy before tissue sampling. We snap-froze all samples using liquid nitrogen, which were stored at -80°C prior to RNA extraction. Ethics Committee of Xinhua Hospital in Shanghai Jiaotong University approved this study.

### Animals

We utilized 4-week-old BALB/c nude mice (n = 20) with 15~20 g weight (SLARC, Shanghai, China). Ethics Committee in Xinhua Hospital of Shanghai Jiaotong University approved animal experiments.

### Cell culture and treatment

We obtained lung cancer cell lines H1975, H1299, PC9, A549, and H1650 along with normal lung epithelial cell line HEAS-2B from American Type Culture Collection (Manassas, VA) and cultured them in Dulbecco’s modified Eagle’s medium (DMEM; Gibco, Gaithersburg, MD) containing fetal bovine serum (FBS; Gibco), 100 IU/mL 10% penicillin, and streptomycin of 100 μg/mL at 37°C and 5% CO_2_. Lentiviral-stabilized hsa_circ_0001869 silenced vector (sh-circRNA), miR-638 inhibitors, miR-638 mimics, lentiviral-stabilized FOSL2 overexpression (LV-FOSL2 or FOSL2) and lentiviral-stabilized FOSL2 silencing (sh-FOSL2) vectors, and negative controls were transfected into cultured A549 or PC9 cells before for other experiments. Hsa_circ_0001869-targeting shRNA, miR-638 mimics, and miR-638 inhibitors were synthesized through Sengong Biotech (Shanghai, China), with sense sequence: sh-circRNA: forward: 5′-CCCUUCAUAACACAAGGCUUU-3′, reverse: 5′-AGCCUUGUGUUAUGAAGGGUU-3′. miR-638 mimic: forward: 5′-AGGGAUCGCGGGCGGGUGGCGGCCU-3′, reverse: 5′-GCCGCCACCCGCCCGCGAUCCCUUU-3′. miR-638 inhibitor: 5′-AGGCCGCCACCCGCCCGCGAUCCCU-3′. We obtained lentiviral-based shRNA targeting hsa_circ_0001869 and lentiviruses overexpressing FOSL2 from GeneChem (Shanghai, China).

### Bioinformatics analysis

We predicted CircRNA/miRNA target genes through https://circinteractome.nia.nih.gov/. We predicted interactions between mRNA and miRNA by http://www.targetscan.org/.

### Colony formation and cell proliferation assays

We assayed cell proliferation with Cell Counting Kit-8 (CCK-8; Invitrogen, Carlsbad, CA). We seeded cells that transfected into plates with 96 wells in triplicate at 2,000 cells per well and assayed cell viability at 0, 1, 2, 3, and 4 d after seeding, following the standard kit procedures. Colony formation was assayed for transfected cells seeded into plates with 6 wells at 2,000 cells/well and grown in DMEM with FBS of 10% for ten days. We counted colonies and photographed them after fixing and staining.

### Flow cytometry cell cycle assay

We fixed cells in ethanol of 70% overnight at 4°C. The cells that fixed were resuspended in a staining solution (Beyotime, Shanghai) and incubated at 4°C for half of an hour. We measured stained cells by flow cytometry (Beckman Coulter).

### Fluorescence *in situ* hybridization (FISH)

We utilized hsa_circ_0001869-specific FITC-labeled probes for FISH [30]. Probes specific to hsa_circ_0001869 (Dig-5′-GATTATCAAGGAAAAGCCCTTCATAACAC-3′-Dig) were used. We counterstained nuclei with 4,6-diamidino-2-phenylindole (DAPI). We finished the procedures following manufacturer’s instructions (Genepharma, Shanghai, China).

### qRT-PCR

We extracted RNA with TRIzol reagent (Invitrogen) and synthesized cDNA using the pTRUEscript First Strand cDNA Synthesis Kit (Aidlab, Beijing, China). We conducted qRT-PCR using 2× SYBR Green qPCR Mix (Invitrogen) through an ABI 7900HT qPCR system (Thermo Fisher Scientific, Waltham, MA, USA). We detected expression fold-change by 2^−ΔΔCT^ method. qRT-PCR amplification was performed using primers: hsa_circ_0001869: forward: 5′-GAGCTGGATTATCAAGG-3′; reverse: 5′-GATCAATGGCGGAATAAGCAG-3′; miR-638: forward: 5′-AAGGGATCGCGGGCGGGT-3′; reverse: 5′-CAGTGCAGGGTCCGAGGT-3′; FOSL2: forward: 5′-GAGAGGAACAAGCTGGCTGC-3′; reverse: 5′-GCTTCTCCTTCTCCTTCTGC-3′; U6: forward: 5′-CTCGCTTCGGCAGCACA-3′; reverse: 5′-AACGCTTCATTTGCGT-3′; GAPDH: forward: 5′-AATCCCATCACCATCTTCC-3′; reverse: 5′-CATCACGCCACAGTTTCC-3′. FOSL2 and hsa_circ_0001869 expression levels were normalized to GAPDH and miR-638 expression level was normalized to U6.

### Transwell assay

For Transwell migration assays, we suspended 2 × 10^4^ cells in 200 μL serum-free culture medium and inserted them to upper chambers. To perform invasion assays, Transwell chambers were precoated using Matrigel (BD Biosciences, San Jose, CA), and we supplied equivalent number of cells to upper chamber. We then added 500 μL DMEM including FBS of 15% to lower chamber. We erased cells in upper chamber after incubation for 1 d, and we fixed cells that invaded or migrated the lower membrane surface with paraformaldehyde of 4% and stained them with crystal violet solution of 0.1%. We photographed and counted migrated or invading cells. 12 fields were counted in migration and invasion assays in each group.

### Luciferase reporter assay

We leveraged pGL3-reporter luciferase vector (Promega, Madison, Wisconsin) to build pGL3-FOSL2 3′UTR, pGL3-hsa_circ_0001869, pGL3-FOSL2 3′UTR-Mut, and pGL3-hsa_circ_0001869-Mut vectors. We seeded 293T cells onto culture plates with 24 wells, cultured them for 1 d, and cotransfected them with Mut or WT FOSL2 3′-UTR/hsa_circ_0001869 and miR-638 mimics or miR-NC following standard protocol. We monitored luciferase activity at 2 d post transfection by Dual-Luciferase Reporter Assay System (Promega, USA). We computed firefly luciferase ratio for each well that transfected.

### Western blot analysis

We lysed tissues or cells, and measured protein concentrations through BCA Protein Assay Kit (Pierce, Rockford, IL). We resolved protein samples via SDS-polyacrylamide gel electrophoresis and transferred them to nitrocellulose membranes. We incubated membranes with anti-FOSL2 and anti-GAPDH (both from Cell Signaling Technology, Beverly, MA). After washing, we incubated membranes with secondary antibodies coupled to horseradish peroxidase for one hour at room temperature. We examined protein signals through Western Lightning Plus ECL kit (PerkinElmer, Waltham, MA, USA) and quantified them through densitometry.

### Tumor xenograft formation and metastasis assay

We injected 2 × 10^7^ viable cells from sh-circRNA A549 cells, LV-FOSL2 A549 cells, or miR-638 inhibitor A549 cells into nude mice right flank. We detected tumor volumes every five days using vernier calipers, and calculated tumor size via formula: volume = 0.5 * width^2^ * length. After 1 m, we euthanatized mice for qRT-PCR analyses.

For metastasis analyses, we transfected sh-circRNA or negative control A549 cells (2 × 10^5^) using luciferase expression vectors, and intravenously injected cells into the mice tails. After 1 m, we detected A549 cell metastasis by bioluminescence imaging after intravenous luciferin (150 mg luciferin/kg body weight) injection into mouse tails.

### Immunohistochemistry (IHC)

We fixed tissue samples in paraformaldehyde of 4%, embedded them in paraffin, and sectioned them. We incubated sections with anti-Ki-67 primary antibodies at 4°C overnight, followed by incubation using HRP-conjugated secondary antibodies.

### Statistical analyses

We evaluated statistical significance through one-way variance analysis and employed Tukey to compare column pairs. We utilized Pearson’s correlation to measure association between two groups via GraphPad Prism (GraphPad, La Jolla, CA, USA). We analyzed cumulative recurrence and survival rates through Kaplan-Meier method. Data are denoted by mean ± SEM. P values < 0.05 were regarded significant.

### Ethics approval

Xinhua Hospital Committee at Shanghai Jiaotong University approved the study.
